# Not Always Asthma: Clinical and Legal Consequences of Delayed Diagnosis of Laryngotracheal Stenosis

**DOI:** 10.1155/2014/325048

**Published:** 2014-12-18

**Authors:** Adam C. Nunn, S. Ali R. Nouraei, P. Jeremy George, Guri S. Sandhu, S. A. Reza Nouraei

**Affiliations:** ^1^Department of Trauma & Orthopaedics, Ipswich Hospital, Heath Road, Ipswich IP4 5PD, UK; ^2^The Neutral Corner Ltd, Clavering House, Clavering Place, Newcastle upon Tyne, NE1 3NG, UK; ^3^Department of Respiratory Medicine, University College Hospital, Euston Road, London NW1 2BU, UK; ^4^Department of Ear, Nose, and Throat Surgery, Chelsea and Westminster Hospital, Fulham Road, London SW10 9NH, UK; ^5^Department of Ear, Nose, and Throat Surgery, The Royal Free Hospital, Pond Street, London NW3 2QG, UK

## Abstract

Laryngotracheal stenosis (LTS) is a rare condition that occurs most commonly as a result of instrumentation of the airway but may also occur as a result of inflammatory conditions or idiopathically. Here, we present the case of a patient who developed LTS as a complication of granulomatosis with polyangiitis (GPA), which was misdiagnosed as asthma for 6 years. After an admission with respiratory symptoms that worsened to the extent that she required intubation, a previously well 14-year-old girl was diagnosed with GPA. Following immunosuppressive therapy, she made a good recovery and was discharged after 22 days. Over subsequent years, she developed dyspnoea and “wheeze” and a diagnosis of asthma was made. When she became pregnant, she was admitted to hospital with worsening respiratory symptoms, whereupon her “wheeze” was correctly identified as “stridor,” and subsequent investigations revealed a significant subglottic stenosis. The delay in diagnosis precluded the use of minimally invasive therapies, with the result that intermittent laser resection and open laryngotracheal reconstructive surgery were the only available treatment options. There were numerous points at which the correct diagnosis might have been made, either by proper interpretation of flow-volume loops or by calculation of the Empey or Expiratory Disproportion Indices from spirometry data.

## 1. Introduction

Laryngotracheal stenosis (LTS) refers to abnormal narrowing of the central airways from the glottic inlet to the carina. The commonest benign and malignant causes of this condition are intubation-related airway stenosis and tracheal squamous cell carcinoma, respectively [1, 2]. Once suspected, LTS can be easily diagnosed with flow-volume loop testing [3] but being a very uncommon cause of exertional dyspnoea and “wheeze,” without an appropriate index of clinical suspicion, its symptoms are frequently mistaken for asthma [4, 5]. This diagnostic delay prolongs morbidity and can lead to the loss of the operability window for tracheal malignancies or end in acute-on-chronic respiratory failure. In the case of benign strictures, delay can reduce the probability of treatment success, with the result that lesions that might otherwise have been suitable for intralesional steroid therapy reach a point where there is no other option except open airway reconstructive surgery. Here, we present the case of a patient with granulomatosis with polyangiitis (GPA) whose airway stenosis was mistaken for asthma for six years and consider its clinical and medicolegal consequences.

## 2. Case Presentation

**Figure 1:**
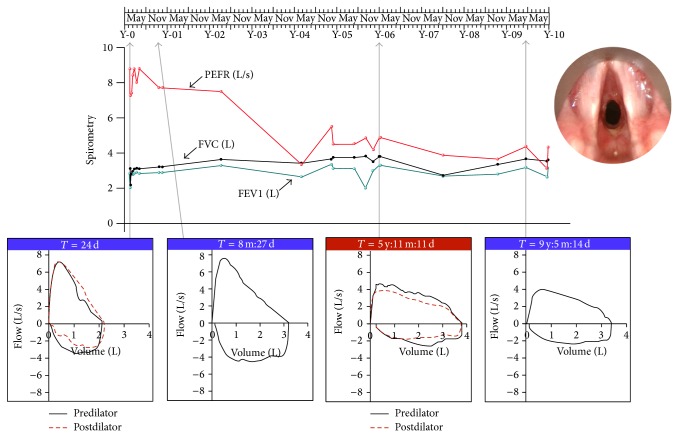
Investigations during the period of mistaken diagnosis. Spirometry and flow-volume loops taken over a ten-year period from initial presentation through to emergency laser resection of the patient's stenosis. The inset image is that of the patient's larynx via an endoscope at the time of her first surgery and shows a Myer-Cotton grade 2 (51–70%) mature fibrotic subglottic stenosis.

A 14-year-old girl, who was previously well, presented with cough, breathlessness, and flu-like symptoms and was initially diagnosed with community-acquired pneumonia. She progressed to respiratory failure and required intubation and mechanical ventilation. She had a diagnostic bronchoscopy with biopsies and pulmonary lavage, a renal biopsy, and serological investigations for anti-nuclear antibodies (ANA) and anti-neutrophil cytoplasmic antibodies (ANCA). She was given a diagnosis of multisystem ANCA-associated vasculitis, likely due to GPA, and received intravenous methylprednisolone and cyclophosphamide and underwent plasmapheresis. She responded well to these therapies and was extubated after eight days and discharged after 22 days. During her admission, she had multiple lung function tests and a flow-volume loop examination, which were all normal (Figure 1).

Following her admission, she received regular follow-up from the respiratory team that had delivered her inpatient care. She remained asymptomatic from a pulmonary perspective for the next four years, until she began to complain of exertional dyspnoea, wheeze, and disturbed sleep due to her breathing difficulties. She was given inhaled bronchodilators and corticosteroids by her general practitioner and the diagnosis of asthma was upheld by her respiratory team over the next 6 years.

During this time, she had a series of lung function tests, including a number of flow-volume loops. At around the time she began to experience symptoms of dyspnoea and “wheeze,” her lung function tests changed, showing a disproportionate reduction in peak expiratory flow rate (PEFR) relative to forced expiratory volume in one second (FEV_1_), a classical sign of laryngotracheal obstruction, which was apparently missed by her doctors (see Figure 1). The Empey Index has been used historically to detect airway obstruction from lung function tests, in which the ratio of FEV_1_ (expressed in mL) to PEFR (in L min^−1^) is taken and a value of >10 is considered as evidence of LTS [6]. At the onset of symptoms, her Empey Index was 13.2. In spite of this, the diagnosis of asthma was not challenged, and she went on to receive different asthma medications but without objective improvement.

She had a further flow-volume loop test approximately six years after her initial presentation and two years after her respiratory symptoms first developed. This showed an unmistakable pattern of upper airway stenosis with minimal reversibility but the patient was given further reassurance about her asthma diagnosis and advice on management at that time. A further opportunity to establish the diagnosis was missed two years later when the patient became concerned that her symptoms might be due to tracheal stenosis and raised these concerns with her respiratory specialist. These concerns were noted and the physician suggested further flow-volume loop testing. Although she then missed two further appointments, subsequent independent review of the case pointed out that a further set of tests were not necessary to confirm that the patient's suspicions were indeed correct.

Approximately eighteen months later, the patient became pregnant, which led to a significant worsening of her symptoms. She was reviewed by the respiratory team who stressed the importance of strict adherence to asthma treatment. Her symptoms worsened and she was admitted with a presumptive diagnosis of preeclampsia and the admitting team was reassured that her asthma was stable. Just over two weeks later, she developed respiratory failure and was diagnosed with presumptive subglottic stenosis by an obstetric anaesthetist. This diagnosis was confirmed by an ENT surgeon and she underwent emergency laser airway surgery for a grade 2 (Myer-Cotton grade; 50–70% obstruction) mature fibrotic subglottic stricture. She has since required multiple endoscopic procedures to maintain her airway and will require open laryngotracheal reconstruction to achieve long-term symptom remission. Given the risks inherent with this surgery, however, she has chosen to postpone it until her children are older.

A claim for clinical negligence was made and the defendants conceded breach of duty of care from the time the patient first developed symptoms. This was based on the fact that patients with GPA are known to be at risk of developing subglottic stenosis [7], that disproportionate reduction in PEFR in relation to FEV_1_ should have raised suspicions of upper, as opposed to lower, airway obstruction [6], and that a reasonable clinician practicing in the field of respiratory medicine ought to have known and acted upon these facts. Damages of £625,000 were awarded based on patient suffering for the duration of mistaken diagnosis and the fact that the delay also led to an unfavourable clinical outcome, from one that could have been satisfactorily managed with intralesional steroid therapy and minimally invasive surgery [8, 9] to one requiring more invasive open cervicomediastinal laryngotracheal surgery.

## 3. Discussion

The presentation of exertional dyspnoea and “wheeze” is a common problem presenting to primary and secondary care, and the majority of cases will be correctly attributed to asthma. The British Thoracic Society recommends that unless an alternative diagnosis appears more likely from the clinical history, those with symptoms suggestive of asthma should be commenced on empirical asthma therapy, and further investigation only pursued in those for whom response is poor [10]. Furthermore, the differentiation of asthma from anatomical causes of dyspnoea and “wheeze” can be extremely challenging. Upper airway obstruction classically produces “stridor”; however, wheeze is almost as commonly the presenting symptom [11].

GPA is a small-vessel vasculitis that presents classically with a combination of upper and lower airway symptoms and renal disease. Upper airway symptoms frequently involve nasal discharge, crusting, ulceration, nasal septum perforation, and, in advanced cases, complete destruction of the nasal septum, resulting in a saddle nose deformity [12]. Lower airway involvement may be mistaken for an infective process or detected as pulmonary infiltrates on chest radiograph. Otological and ophthalmic manifestations are also common, such as serous otitis media or conjunctivitis, episcleritis, scleritis, or uveitis, as are oral lesions such as ulcers or gingival hyperplasia. Laryngeal stenosis is not uncommon in patients with GPA (6–25%) and may precede other organ system involvement [13]. In these patients, inflammation of the subglottic mucosa eventually leads to scarring and fibrosis, resulting in stenosis of the tracheal lumen. Unfortunately, some patients experience recurrent airway stenosis even after symptoms in other organ systems have been successfully treated.

Other potential mimics of asthma can be found at virtually every level of the respiratory system (see Table 1). At the level of the larynx, vocal cord dysfunction can cause inappropriate adduction of the cords during inspiration producing wheeze and dyspnoea, or in those who have previously been artificially ventilated subglottic stenosis may produce these symptoms (even many years after the event). Below the level of the larynx, intrinsic tumours of the trachea or mediastinal tumours that compress the airways can produce a similar clinical picture. Congenital defects of the airway may also mimic asthma, such as a subglottic web or abnormalities of the great vessels that compress the trachea, which may be haemodynamically insignificant (these patients may also complain of dysphagia) [14, 15]. Interestingly, foreign bodies may also cause adult-onset respiratory symptoms even when the patient has no recollection of an aspiration event [16]. More common asthma mimics include gastroesophageal reflux, which can cause bronchospasm, and congestive cardiac failure (so-called “cardiac wheeze”).

Although clinically a difficult diagnosis, there are a number of investigations that aid the differentiation of asthma from localised upper airway obstruction. Flow-volume loops show a classical picture, with flattening of both the inspiratory and expiratory limbs, but are difficult to obtain in nonspecialist settings. As a consequence, the Empey Index or a refined version termed the “Expiratory Disproportion Index” (EDI; FEV_1_ [L]/PEFR [L^−S^] × 100) has been developed to allow the diagnosis to be inferred from standard spirometry and peak expiratory flow data. At a threshold of >50, the EDI is a highly sensitive and specific tool for establishing the diagnosis of LTS [17].

In this case, merely considering alternative diagnoses, thoroughly examining the results of ordered tests (particularly the flow-volume loops), and recognising two key risk factors from the clinical history, previous intubation and a history of GPA, may have prevented this unfortunate and costly mistake for the parties involved. However, LTS is a very uncommon cause of a very common clinical presentation (exertional dyspnoea, wheeze, and reduced PEFR). This diagnostic difficulty is further amplified in situations where airway stenosis arises insidiously such as tracheal tumours or idiopathic subglottic stenosis. Upper airway stenosis associated with GPA also arises insidiously and without heralding signs, but in a patient group known to be at risk of this complication [7]. Owing to the time scale of events in this patient's case (with the stricture developing four years after intubation), the diagnosis was felt most likely to be a complication of her GPA rather than a consequence of intubation. There were several points at which the opportunity to establish the diagnosis was missed, but significantly the point in time from which breach of duty of care was admitted was from the time when symptoms were first reported and upper airway obstruction became evident on standard lung function testing.

## 4. Conclusion

Mistaken and delayed diagnosis of LTS is a frequent occurrence, with as many as 10% of patients with this condition remaining undiagnosed for ten or more years [4, 5, 11]. This delay leads to prolonged respiratory morbidity, can put patients at ongoing risk of acute-on-chronic respiratory failure, and can render a tracheal cancer inoperable. It can be largely eliminated by maintaining an appropriate index of clinical suspicion and through a simple examination of standard lung function data for disproportionate changes in PEFR in relation to the FEV_1_ using the Empey Index or EDI, particularly if the patient fails to respond to initial asthma therapy. This case emphasises the importance of maintaining clinical vigilance to prevent morbidity and patient suffering, and that failure to act on clinical findings or investigations amounts to a breach of duty of care on the part of the treating physician and can lead to a successful claim for clinical negligence.

## 5. Learning Points

1. Suspect upper airway causes of dyspnoea and “wheeze” in any patient who has recalcitrant asthma, particularly if they have risk factors for airway stenosis such as smoking, GPA, or a history of previous intubation.
2. Upper airway obstruction may present with respiratory sounds that are clinically indistinguishable from wheeze.
3. Cardiac failure and GORD are also common “asthma mimics.”
4. Calculate the Empey Index or Expiratory Disproportion Index for spirometry data in all patients in whom this investigation is performed, to ensure that upper airway causes of respiratory symptoms are not missed.

## Figures and Tables

**Table 1:** Major differentials for adult-onset asthma and their associations.

Anatomical location	Differential diagnoses	Associations
Larynx	Vocal cord dysfunction	Paradoxical movement: anxiety, gastroesophageal reflux Paralysis: previous neck surgery, intubation
	Arytenoid cartilage fixation	Rheumatoid arthritis, granulomatosis with polyangiitis
	Subglottic web	Tuberculosis, sarcoidosis

Trachea and main stem bronchi	Primary tumours of the airway Squamous cell carcinoma^*^ Endobronchial carcinoid^†^	SCC: smoking
	Strictures	Intubation, tracheotomy, direct trauma Female gender (idiopathic subglottic stenosis) Vasculitis, particularly granulomatosis with polyangiitis
	Extrinsic compression of the airway Mediastinal tumours Encircling vascular anomalies Lymphadenopathy (e.g., sarcoidosis) Retrosternal goiter	

Bronchi	Foreign body	Children, those with neurological deficits
	Gastroesophageal reflux disease	Various—obesity in particular

Alveoli	Congestive cardiac failure	Older age, orthopnoea, paroxysmal nocturnal dyspnoea, and dependent oedema

^*^Most common tumour of the trachea.

^†^May produce wheeze through an endocrine mechanism.

## Abbreviations

GPA: Granulomatosis with polyangiitis
ANA: Anti-nuclear antibodies
ANCA: Anti-neutrophil cytoplasmic antibodies
PEFR: Peak expiratory flow rate
FEV_1_: Forced expiratory volume in 1 second
SCC: Squamous cell carcinoma
EDI: Expiratory disproportion index.
